# Whole-transcriptome analyses identify key differentially expressed mRNAs, lncRNAs, and miRNAs associated with male sterility in watermelon

**DOI:** 10.3389/fpls.2023.1138415

**Published:** 2023-03-02

**Authors:** Zhen Yue, Xiaona Pan, Jiayue Li, Fengfei Si, Lijuan Yin, Yinjie Hou, Xiaoyao Chen, Xin Li, Yong Zhang, Jianxiang Ma, Jianqiang Yang, Hao Li, Feishi Luan, Wenfeng Huang, Xian Zhang, Li Yuan, Ruimin Zhang, Chunhua Wei

**Affiliations:** ^1^ State Key Laboratory of Crop Stress Biology in Arid Areas, College of Horticulture, Northwest A&F University, Yangling, Shaanxi, China; ^2^ College of Horticulture and Landscape Architecture, Northeast Agricultural University, Harbin, Heilongjiang, China; ^3^ Vegetable Research Institute of Hainan Academy of Agricultural Sciences, Haikou, Hainan, China; ^4^ State Key Laboratory of Vegetable Germplasm Innovation, Tianjin, China; ^5^ College of Horticulture Science and Engineering, Shandong Agricultural University, Tai’an, Shandong, China

**Keywords:** watermelon, male sterility, lncRNAs, miRNAs, regulatory network

## Abstract

Male sterility is a valuable trait for watermelon breeding, as watermelon hybrids exhibit obvious heterosis. However, the underlying regulatory mechanism is still largely unknown, especially regarding the related non-coding genes. In the present study, approximately 1035 differentially expressed genes (DEGs), as well as 80 DE-lncRNAs and 10 DE-miRNAs, were identified, with the overwhelming majority down-regulated in male-sterile floral buds. Enrichment analyses revealed that the general phenylpropanoid pathway as well as its related metabolisms was predicted to be altered in a mutant compared to its fertile progenitor. Meanwhile, the conserved genetic pathway *DYT1*-*TDF1*-*AMS*-*MS188*-*MS1*, as well as the causal gene *ClAMT1* for the male-sterile mutant Se18, was substantially disrupted during male reproductive development. In addition, some targets of the key regulators *AMS* and *MS188* in tapetum development were also down-regulated at a transcriptional level, such as *ABCG26* (*Cla004479*), *ACOS5* (*Cla022956*), *CYP703A2* (*Cla021151*), *PKSA* (*Cla021099*), and *TKPR1* (*Cla002563*). Considering lncRNAs may act as functional endogenous target mimics of miRNAs, competitive endogenous RNA networks were subsequently constructed, with the most complex one containing three DE-miRNAs, two DE-lncRNAs, and 21 DEGs. Collectively, these findings not only contribute to a better understanding of genetic regulatory networks underlying male sterility in watermelon, but also provide valuable candidates for future research.

## Introduction

Male sterility is a common phenomenon in flowering plants that has been widely applied in crop hybrid seed production and heterosis utilization. As a complex biological process, numerous transcription factors have been validated to precisely regulate the development of male reproductive organs (i.e., anthers), such as the tapetum-specific genetic pathway *DYT1*-*TDF1*-*AMS*-*MS188*-*MS1* (*MS188* is also known as *MYB103*/*MYB80*) (Wilson et al., 2001; Sorensen et al., 2003; Zhang, 2006; Zhang et al., 2007; Zhu et al., 2008; Lu et al., 2020). In this regulatory cascade, *DYT1* (*DYSFUNCTIONAL TAPETUM 1*) and *AMS* (ABORTED MICROSPORES) belong to the bHLH transcription factor (TF) family, *TDF1* (*TAPETAL DEVELOPMENT AND FUNCTION 1*) and *MS188* (*MALE STERILE 188*) encode R2R3 MYB TFs, and *MS1* (*MALE STERILITY 1*) is a TF with leucine zipper-like and PHD-finger motifs. Moreover, proteins encoded by *AMS* and *MS188* functioning as master regulators can activate a series of downstream targets to coordinate pollen development, e.g., *MALE STERILE 2* (*MS2*), *ACYL-COA SYNTHETASE 5* (*ACOS5*), CYTOCHROME P450 genes *CYP703A2* and *CYP704B1*, *POLYKETIDE SYNTHASE A* (*PKSA*) and *PKSB*, *TETRAKETIDE a-PYRONE REDUCTASE1* (*TKPR1*) and *TKPR2* (Xu et al., 2014; Wang et al., 2018; Lu et al., 2020). It is worth mentioning that a similar regulatory pathway has also been verified in tomato and rice pollen development, indicating this is a highly conserved signaling cascade in plants (Jeong et al., 2014; Nan et al., 2017).

Beyond protein-coding genes, non-coding RNAs (ncRNAs) including microRNAs (miRNAs), small interfering RNAs (siRNAs), circular RNAs (circRNAs), and long non-coding RNAs (lncRNAs), have also been confirmed to participate in various aspects of plant growth and development, such as stress-related responses (Hu et al., 2021; Ren et al., 2021; Shi et al., 2021; Wang et al., 2021) and male reproductive development (Li et al., 2015; Yuan et al., 2020). As endogenous non-coding RNAs of 21–25 nt in length, miRNAs mainly contribute to post-transcriptional regulation of downstream targets through mRNA cleavage. For example, *miR156*, *miR159*, and *miR396* can participate in flower development and male fertility in plants, *via* regulating their targets (Millar and Gubler, 2005; Wang et al., 2009; Yuan et al., 2020). With the development of high-throughput sequencing technology, series of differently expressed miRNAs related to male organ development have been identified in various species (Nie et al., 2018; Dhaka et al., 2020). Compared to small miRNAs, lncRNAs are a type of non-coding transcript exceeding 200 nt, and they can be further classified into intergenic lncRNAs (lincRNAs), intronic lncRNAs, and antisense lncRNAs based on their chromosomal locations (Li et al., 2019a; Gao et al., 2020). To date, several lncRNAs have been characterized to function in pollen fertility, such as the lincRNA *BcMF11* in Chinese cabbage (Song et al., 2013) and LDMAR in hybrid rice (Ding et al., 2012). Moreover, approximately 865 differentially expressed lncRNAs were detected to possibly be involved in cotton anther development (Li et al., 2019a). Similarly, potential lncRNAs participating in flower development have also been identified in plants, such as tomato (Yang et al., 2019), strawberry (Kang and Liu, 2015), *Brassica campestris* (Liang et al., 2019), and cotton (Nie et al., 2018; Li et al., 2019a). Interactions among mRNAs, lncRNAs, and miRNAs have revealed that lncRNAs acting as miRNA sponges can competitively bind with miRNAs to indirectly influence the expression of corresponding targets, raising the competitive endogenous RNA (ceRNA) hypothesis (Salmena et al., 2011; Li et al., 2019b; Sun et al., 2020). For example, in watermelon, ceRNA networks of lncRNA/circRNA–miRNA–mRNA interactions, including 23 potential lncRNA–miRNA–mRNA and 125 potential circRNA–miRNA–mRNA interactions, were constructed in response to CGMMV infection (Sun et al., 2020). In maize, ceRNA regulatory networks consisting of 51 known miRNAs, 28 potentially novel miRNAs, 619 ceRNA–miRNA pairs, and 869 miRNA–target gene pairs were proposed to play roles during anther development (Li et al., 2019b). Similarly, correlation networks of lncRNA/circRNA–miRNA–mRNA interactions were inferred to act during flower development in tomato (Yang et al., 2019) and *Brassica campestris* (Liang et al., 2019), respectively.

Watermelon is grown worldwide, and its hybrids exhibit obvious heterosis. Traditionally, the commercial hybrid seeds of watermelon are mainly produced by artificial emasculation and pollination, requiring lots of time and labor. However, the application of male-sterile lines could sufficiently overcome these constraints of hybrid production. To date, several male-sterile mutants have been reported in watermelon (Wei et al., 2021; Zhang et al., 2021), and only one causal gene, *ClATM1*, has been functionally characterized from the male-sterile mutant Se18 previously (Zhang et al., 2021). Compared to the wild type *ClATM1*, its mutant allele contains a 10-bp deletion in the second exon that results in a truncated protein without the bHLH interaction and functional (BIF) domain, leading to the male sterile phenotype in Se18 (Zhang et al., 2021). However, the underlying regulatory network is still poorly understood. In this study, using high-throughput sequencing, we identified differentially expressed genes (DEGs), DE-lncRNAs, and DE-miRNAs at a genome-wide scale from male floral buds between mutant Se18 and its fertile wild-type progenitor. Comparative analyses revealed that the phenylpropanoid-related metabolisms, as well as the highly conserved genetic regulatory pathway *DYT1*-*TDF1*-*AMS*-*MS188*-*MS1*, were predicted to be disrupted during male reproductive development. Finally, the corresponding ceRNA networks were constructed, providing a foundation for elucidating the complex underlying mechanisms for male sterility in watermelon.

## Materials and methods

### Plant materials

As described in our previous studies (Wei et al., 2021; Zhang et al., 2021), watermelon mutant Se18 is completely male sterile, and exhibits distinct cytological defects in floral buds at 2.0–2.5 mm in diameter, but without obvious phenotypic differences compared to its fertile wild-type (WT) progenitor. Hence, to precisely investigate the underlying regulatory network associated with male sterility, floral buds with a length from 2.0 to 2.5 mm were independently sampled from mutant Se18 and WT plants to generate male sterile (Ms) and male fertile (Mf) pools, with three replicates respectively. All the plant materials were grown in the farms of Northwest A&F University, Yangling, China, under natural conditions.

### Library preparation and Illumina sequencing

The total RNA were independently extracted from Ms and Mf pools and subsequently sent to Novogene Bioinformatics Technology Co., Ltd. (Beijing, China) for high-throughput sequencing. The chain-specific libraries were constructed for mRNA and lncRNAs, by using the Illumina NovaSeq6000 platform to harvest 150-bp paired-end reads (PE150). The clean data of six libraries (ms_1, ms_2, ms_3 and mf_1, mf_2, mf_3) were mapped onto the watermelon reference genome (97103, V1) using HISAT2 with the parameter ‘–rna-strandness RF’. Then, the software tools StringTie and Cuffmerge were used to assemble the transcripts.

The small RNA libraries were constructed with the corresponding Ms and Mf pools, and deep sequencing was performed on an Illumina Hiseq™ 2500 platform (Novogene, Beijing, China) to generate 50-bp single-end reads. Clean data were obtained after removing reads containing poly-N runs (N% > 10%), reads with 5′ adapter contaminants, reads without a 3′ adapter or the insert tag, and reads containing poly-A, -T, -G, or -C runs, as well as low-quality reads, from raw data. Then, the clean data from the six libraries (ms_1, ms_2, ms_3 and mf_1, mf_2, mf_3) were mapped onto the watermelon reference genome (97103, V1) using Bowtie (Langmead and Salzberg, 2012), to analyze their expression and chromosome distribution.

### Identification of differentially expressed mRNAs, lncRNAs and miRNAs

Based on the fragments per kilobase of transcript per million mapped reads (FPKM), genes with expression changes more than twofold (*q*-value <0.05) were recognized as differentially expressed genes (DEGs). Using BlastP (e-value = 1e-10), all the DEGs were searched against the *Arabidopsis* protein database (TAIR10) to identify their closest homologs/orthologs. Moreover, transcription factors were also annotated according to the plant transcription factor database PlantTFDB (Jin et al., 2017).

To identify lncRNAs at a genome-wide scale in watermelon, the tools CPC2, CNCI, and PFAM were employed to estimate the coding potential of the assembled transcripts (length > 200 nt, FPKM ≥ 0.5). According to the class codes annotated by the program Cuffcompare, the identified lncRNAs were classified into three types, i.e., intergenic, intronic, and antisense lncRNAs. Similarly, based on the FPKM method, lncRNAs with expression changes more than twofold (*q*-value <0.05) were recognized as differentially expressed lncRNAs (DE-lncRNAs).

Using miRBase as a reference (specific species *Arabidopsis thaliana*), the mapped small RNA tags were used to identify known miRNAs. For highly accurate identification, only those small RNA tags perfectly matched with known miRNAs were considered as mature sequences of known miRNAs. After removing tags originating from rRNA, tRNA, snRNA, and snoRNA sequences, the remaining small RNA tags were used to identify novel miRNAs with miREvo (Wen et al., 2012) and miRDeep2 (Friedländer et al., 2012). Based on transcripts per million reads (TPM), DESeq was used to identify differentially expressed miRNAs (DE-miRNAs, including known and novel miRNAs) according to the criteria |log_2_(FC)| ≥ 1 and *p*-adjust < 0.05. The identified miRNAs with a low total read count number (less than 10) in six libraries were discarded. The chromosome distribution of DEGs, DE-lncRNAs, and DE-miRNAs was visualized using Circos.

### Gene ontology and Kyoto encyclopedia of genes and genomes enrichment analysis

To predict the potential functions of lncRNAs, genes located around 100 kb up-stream and down-stream of the DE-lncRNAs were selected as potential *cis*-targets (Ren et al., 2021; Shi et al., 2021). The online tool psRNAtarget and the program psRoot were utilized to predict the potential target genes of DE-miRNAs (Wu et al., 2012; Dai et al., 2018). The subsequent Gene Ontology (GO) and Kyoto Encyclopedia of Genes and Genomes (KEGG) enrichment analyses of DEGs, as well as potential targets of DE-lncRNAs and DE-miRNAs, were performed using OmicShare tools, a free online platform for data analysis (https://www.omicshare.com). Heatmaps were generated using TBtools (Chen et al., 2020).

### Construction potential networks among DEGs, DE-lncRNAs, and DE-miRNAs

To infer the potential regulatory relationship between miRNAs and lncRNAs, mature sequences of DE-miRNAs were submitted to the online tool psRNAtarget as templates to predict their target DE-lncRNAs, with default parameters except for a strict Expectation value of 4. The interaction network among DEGs, DE-lncRNAs, and DE-miRNAs was graphically generated by Cytoscape (Shannon et al., 2003).

### Validation of DEGs, DE-lncRNAs, and DE-miRNAs by qRT-PCR

To validate the expression of DEGs and DE-lncRNAs, total RNA was extracted from Ms and Mf pools using Trizol^®^ reagent, and the first-strand cDNA was subsequently synthesized *via* the FastKing RT Kit with gDNase (TIANGEN, Beijing, China). Specific primers were designed, and the housekeeping gene *ClACT* (*Cla007792*) was used as an internal reference (Wei et al., 2019; Yue et al., 2021). For DE-miRNAs validation, cDNAs were synthesized *via* the miRcute Plus miRNA First-Strand cDNA Kit, and qRT-PCR was conducted using the miRcute Plus miRNA qPCR Kit (TIANGEN, Beijing, China). The forward primers were designed based on miRNA-specific sequences, and the universal reverse primer was contained in the qPCR kit. The housekeeping miRNA was U6 snRNA. All the amplification experiments were performed on a StepOnePlus Real-Time PCR system (Applied Biosystems, Foster City, CA, USA). The relative expression level was calculated using the 2^−ΔΔCt^ method as described in our previous studies (Wei et al., 2019; Yue et al., 2021), and significance was determined by Student’s *t*-test. All the primers used are listed in Supplementary **Supplementary Table 1**.

## Results

### Overview of the whole-transcriptome sequencing dataset

Compared to WT individuals, the male-sterile mutant Se18 showed distinct cytological defects in developmental floral buds with a length from 2.0 to 2.5 mm, such as irregularly shaped and smaller tapetal cells (Wang et al., 2020; Wei et al., 2021). To further uncover the regulatory mechanisms underlying the development of male sterility in watermelon, the corresponding floral buds were independently sampled from Se18 and WT plants and designated as ms_1, ms_2, and ms_3 for male sterility and mf_1, mf_2, and mf_3 for male fertility, respectively, which were subsequently subjected to whole-transcriptome sequencing. The strong correlations among biological replicates suggested that the sequencing data were highly reliable (**Supplementary Figure 1A**). After filtering the raw data, at least 13.00 Gb of clean data were obtained for each sample (**Supplementary Table 2**), with Q30 values higher than 91.10% and more than 81.00% of reads successfully mapped to the reference genome 97103 (V1). Following the filter criteria (**Supplementary Figure 2A**), 36,873 lncRNAs were finally identified (**Supplementary Figure 2B**), with the majority being lincRNAs (**Supplementary Figure 2C**). Compared to protein-coding transcripts (mRNAs), most lncRNAs were characterized to have no more than two exons and shorter sequence lengths (**Supplementary Figures 2D, E**).

**Figure 1:**
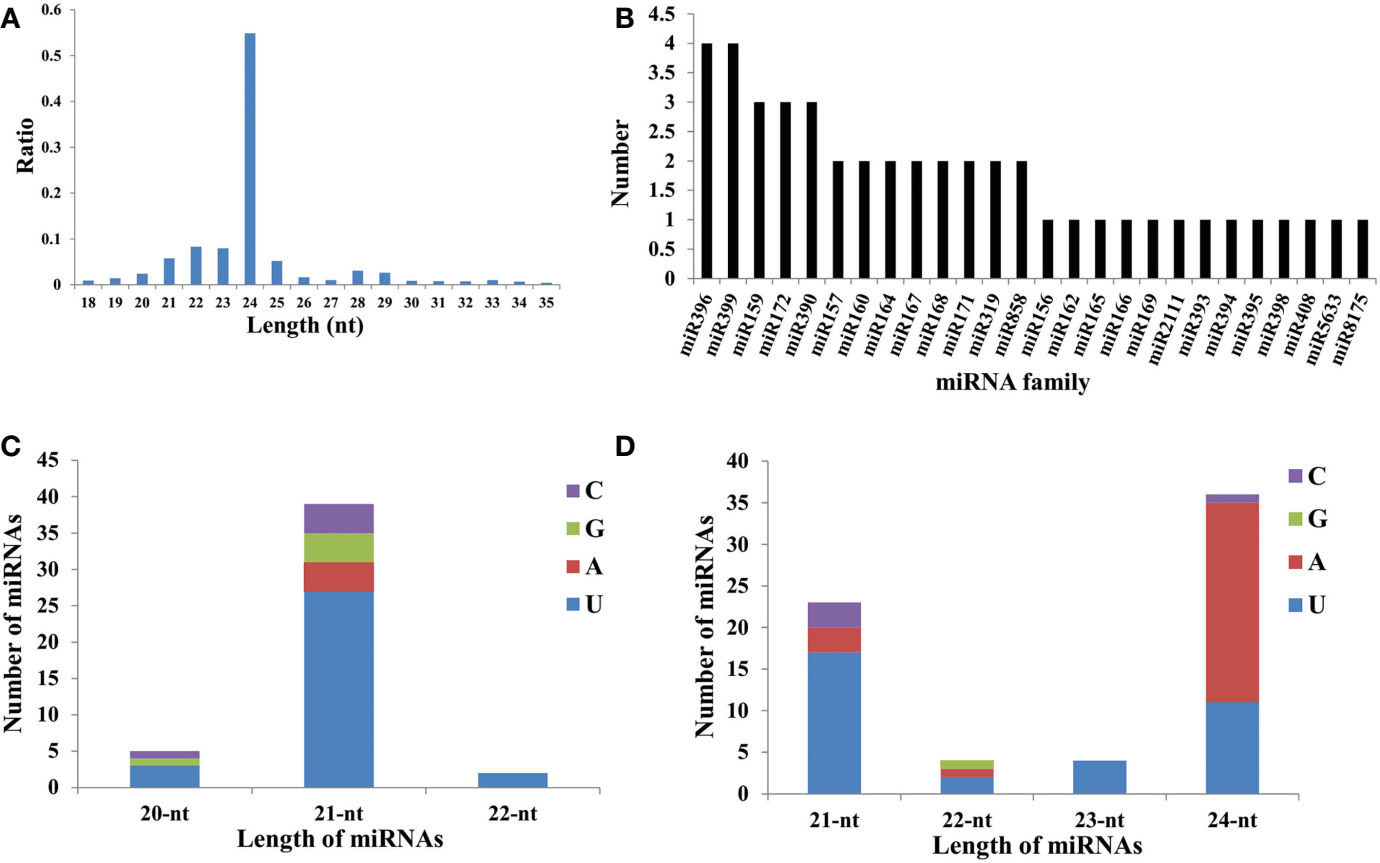
Detailed information for the identified miRNAs. **(A)** The length distribution of small RNA read sequences. **(B)** The member statistics in known miRNA families. The length distribution of known miRNAs **(C)** and novel miRNAs **(D)**. The four nucleotides (C, G, A, and U) are represented in purple, green, red, and blue respectively.

To detect the responsive sRNAs involved in male sterility in watermelon, six libraries were correspondingly constructed for sequencing, obtaining approximately 300 Mb of clean data for each sample with a mapping ratio higher than 88.0% (**Supplementary Table 3**). The reliability of sequencing data among biological replicates was assessed using the Pearson correlation coefficient (**Supplementary Figure 1B**). Further analysis revealed that the size of clean reads mapped onto the genome mainly ranged from 21 to 25 nt, with 24 nt representing the largest class occupying more than 50% in each library (**Figure 1A**), which is similar to the proportions observed in other plants, such as *Arabidopsis* and cotton (Fahlgren et al., 2010; Xing et al., 2014; Yu et al., 2020). Using the database miRBase20.0, a set of 26 known miRNA families were identified and contained approximately 46 members, with the largest two families miRNA396 and miRNA399 harboring four isoforms respectively (**Figure 1B**, **Supplementary Table 4**). Notably, 39 out of the 46 known miRNAs were 21-nt miRNAs, and the majority (69.23%) contained ‘U’ at the first position (**Figure 1C**, **Supplementary Table 4**). Additionally, 67 novel miRNAs were also identified using miREvo and mirDeep2 with default parameters (**Figure 1D**, **Supplementary Table 5**), with the 24-nt miRNAs as the most abundant class (53.73%).

### Differential expression analyses of mRNAs, lncRNAs, and miRNAs

#### Analysis of DEGs

**Figure 2:**
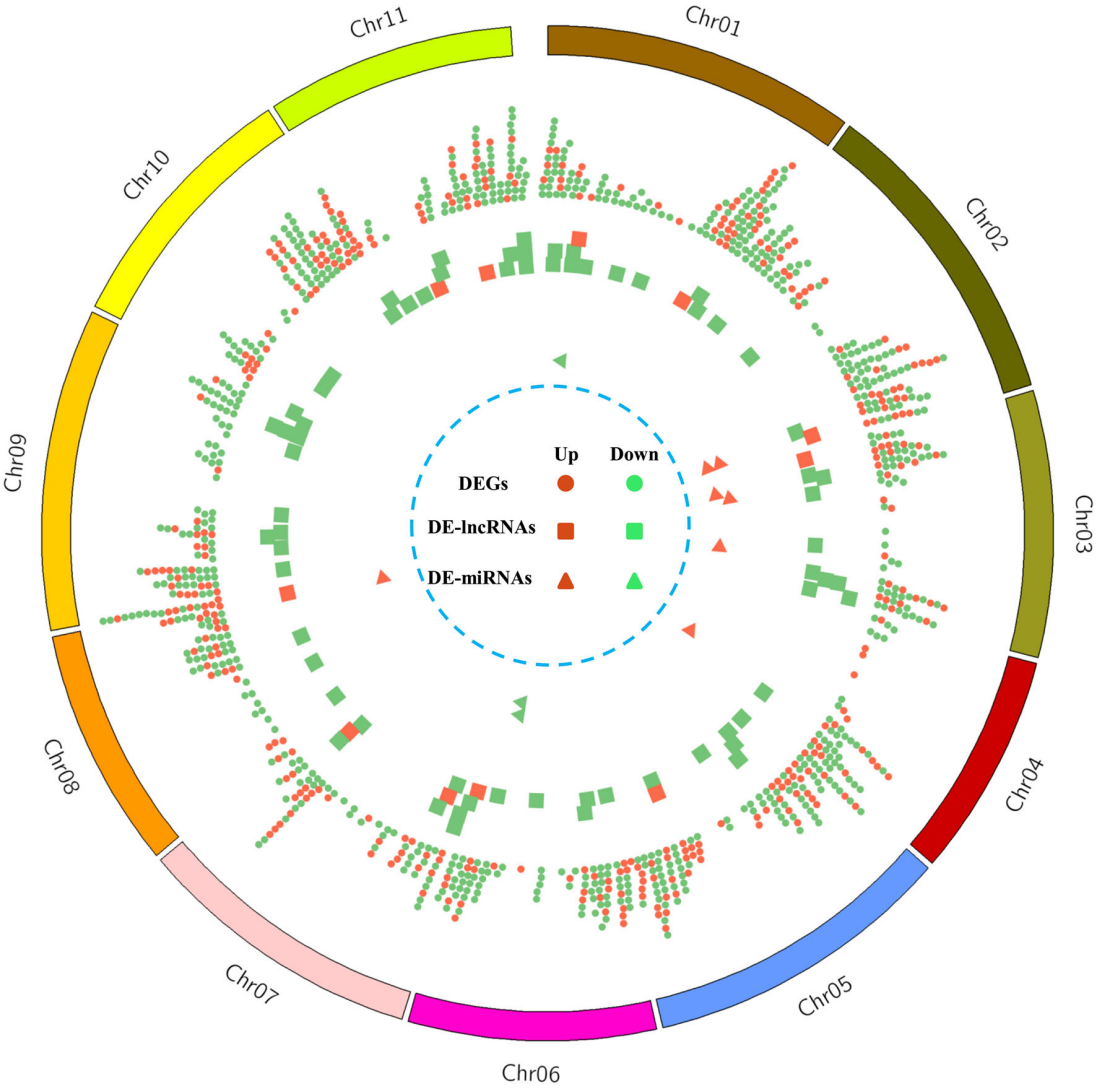
The chromosomal distribution of DEGs, DE-lncRNAs, and DE-miRNAs. From the outside to inside, the circles are watermelon chromosomes, distribution of the up-regulated (red) and down-regulated (green) DEGs (dot), DE-lncRNAs (square), and DE-miRNAs (triangle).

Among all the detected mRNAs, a total of 1035 DEGs were identified, containing 350 up-regulated and 685 down-regulated genes in the male-sterile mutant Se18 (**Figure 2**). Referring to the proteomic data published previously (Wei et al., 2021), about 164 DEGs were also detected with significant changes at the protein level (**Supplementary Table 6**). Moreover, there were three DEGs (*Cla013360*, *Cla013805*, and *Cla021282*) with increased abundance at the transcription level, but reduced protein accumulation in Se18 (**Supplementary Figure 3A**). To further predict the potential function, all 1035 DEGs were searched against the *Arabidopsis* protein database using BlastP. Thus, 961 DEGs were successfully matched with their best homologies (**Supplementary Table 6**), including the functionally characterized tapetum-specific genes *DYT1* (*Cla010083*), *AMS* (*Cla015818*), *TDF1* (*Cla019144*), and *bHLH091* (*Cla010576*).

The GO functional categorization analysis revealed that approximately 266 DEGs, including 68 up-regulated and 198 down-regulated genes, were significantly enriched into 39 GO terms across the three categories biological process (BP, 22), cellular component (CC, 2), and molecular function (MF, 15) (**Supplementary Table 7**). Notably, 18 out of the 22 BP GO terms ultimately corresponded to four terms, ‘stilbene biosynthetic process’ (GO:0009811), ‘lignin biosynthetic process’ (GO:0009809), ‘coumarin biosynthetic process’ (GO:0009805), and ‘pollen wall assembly’ (GO:0010208) (**Supplementary Figures 3B**, **4**). Moreover, KEGG enrichment analysis showed that 55 up-regulated and 112 down-regulated DEGs were classified into 19 different pathways at a *P*-value ≤ 0.05 threshold (**Supplementary Table 8**). The top five significantly enriched metabolic pathways were as follows: Biosynthesis of secondary metabolites (ko01110; 79, 47.30%), Flavonoid biosynthesis (ko00941; 8, 4.79%), Phenylpropanoid biosynthesis (ko00940; 21, 12.57%), Metabolic pathways (ko01100; 113, 67.66%), and Galactose metabolism (ko00052; 9, 5.39%). Taken together, these enrichment analyses suggested that the phenylpropanoid pathway, as well as the related metabolisms, was predicted to be altered in mutant Se18, which is consistent with our previously published results (Wei et al., 2021).

#### Analysis of DE-lncRNAs

To uncover the lncRNAs potentially involved in watermelon male sterility, using parameters |log_2_(FC)| ≥ 1 and *q*-value < 0.05 as thresholds, a total of 80 DE-lncRNAs were identified (**Figure 2**, **Supplementary Table 9**), including 11 up-regulated and 69 down-regulated lncRNAs in mutant Se18. Following published studies (Ren et al., 2021; Shi et al., 2021), we characterized the genes that are *cis*-targets of the DE-lncRNAs, which are located around 100 kb up-stream and down-stream of the DE-lncRNAs. After removing redundant targets, a set of 1247 genes was obtained, comprising 1345 mRNA–DE-lncRNA pairs (**Supplementary Table 10**). To further elucidate the potential functions of DE-lncRNAs, GO enrichment analysis was performed with the 1247 *cis*-target genes, revealing that only 50 genes were significantly categorized into 8 MF and 11 BP GO terms (**Supplementary Table 7**). Notably, the terms ‘phenylalanine ammonia-lyase activity’ (GO:0045548) in MF and ‘L-phenylalanine catabolic process (GO:0006559) in BP were enriched, suggesting the potential functions of the related DE-lncRNAs in the phenylalanine metabolic process. Additionally, KEGG pathway enrichment analysis showed that only 33 targets were enriched in six different pathways (**Supplementary Table 8**), including the phenylalanine metabolism (ko00360; 9, 27.27%), in line with the GO analysis.

Combined with the RNA-seq data, approximately 78 out of the 1247 *cis*-target genes were identified as DEGs (**Supplementary Table 10**), including eight transcription factors such as *Cla002749* (bHLH), *Cla009235* (WRKY), and *Cla021810* (MYB). GO functional categorization analysis revealed that only 7 up-regulated and 13 down-regulated *cis*-targets were significantly enriched in approximately 32 GO terms (**Supplementary Table 7**). Notably, the MF terms were mainly related to protein methyltransferase activity, such as histone-lysine *N*-methyltransferase activity (GO:0018024) and protein-lysine *N*-methyltransferase activity (GO:0016279), while BP terms were involved in the lysine catabolic process (e.g., terms GO:0006554, GO:0006553, GO:0009068), terpenoid biosynthetic process (e.g., terms GO:0016104, GO:0006722, GO:0019742), and lipid metabolic process (e.g., terms GO:0006629, GO:0044255) (**Supplementary Figures 3C, D**). Consistently, KEGG enrichment analysis showed that terpenoid backbone biosynthesis (ko00900) and glycerolipid metabolism (ko00561) were also significantly enriched (**Supplementary Table 8**), inferring that lncRNAs might regulate genes involved in synthesis and modification of amino acids, as well as the lipid metabolic process.

#### Analysis of DE-miRNAs

To identify the potential miRNAs involved in male sterility, expression of all the detected miRNAs was estimated by the TPM approach and subsequently compared between WT and mutant Se18 sequence data. As a result, only 10 DE-miRNAs were obtained (**Figure 2**, **Supplementary Table 11**), including four known and six novel miRNAs. Among the known miRNAs, both of the miR396 members were down-regulated in the male-sterile mutant and predicted to regulate more than 200 targets (**Supplementary Table 11**). However, only about ten targets were identified as DEGs according to the RNA-seq data. In contrast, five out of six novel DE-miRNAs were up-regulated in Se18, with dozens of targets but only few characterized as DEGs. Additionally, 18 DE-lncRNAs were predicted as potential targets of 30 miRNAs, but only three were recognized as DE-miRNAs (**Supplementary Table 12**).

### Validation of DE-mRNAs, DE-miRNAs, and DE-lncRNAs by qRT-PCR

**Figure 3:**
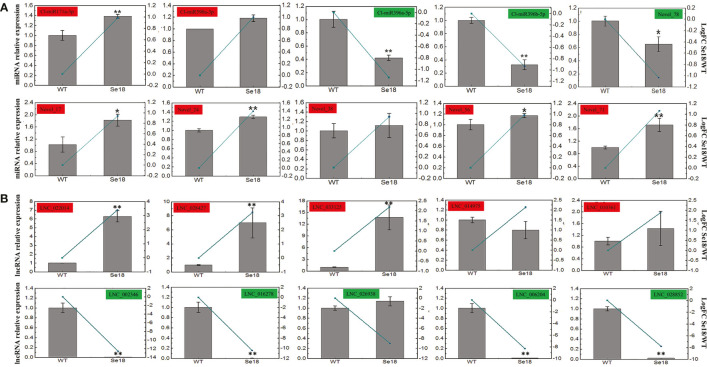
Expression validation of DE-miRNAs **(A)** and DE-lncRNAs **(B)** by qRT-PCR. Gray bars and green lines represent the relative expression and bioinformatics results respectively. The relative expression levels of each gene in WT are normalized as reference and presented as means ± SD (three biological replicates, *P < 0.05, **P < 0.01, Student’s *t*-test).

To validate the differential expression of mRNAs, lncRNAs, and miRNAs based on the multi-omics analysis, qRT-PCR was utilized to confirm the expression patterns between male fertile and sterile buds. As a result, the transcriptional levels of ten selected DEGs were consistent with the bioinformatic analysis (**Supplementary Table 13**), which have also been confirmed to match with their protein accumulations in our previous published research (Wei et al., 2021). Moreover, expression patterns of all ten DE-miRNAs and ten randomly selected DE-lncRNAs were generally concordant with the bioinformatic results (**Figure 3**), suggesting the reliability of the high-throughput sequencing data.

### Construction of miRNA–lncRNA–mRNA regulatory networks

**Figure 4:**
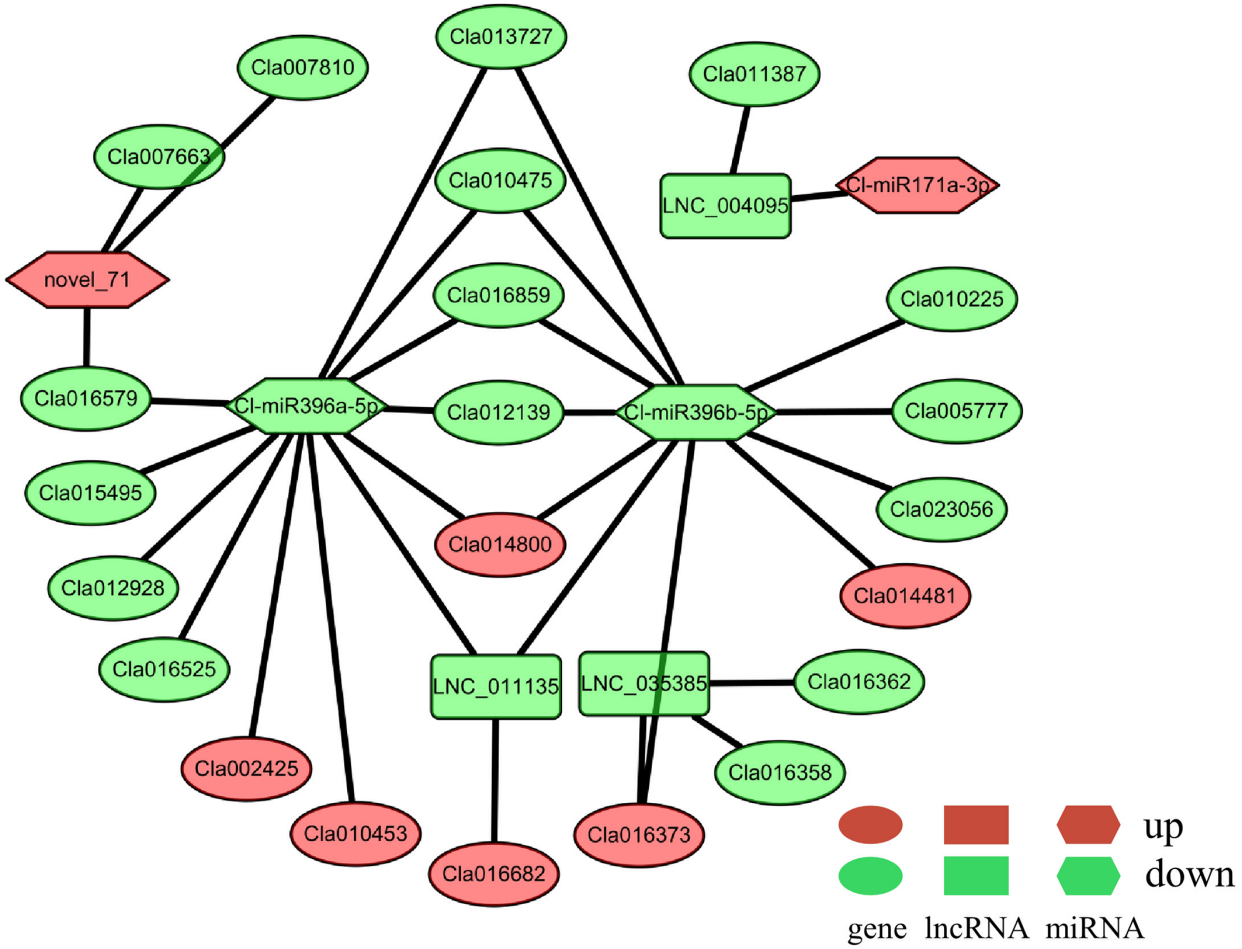
ceRNA regulatory networks predictably involved in male sterility of watermelon. DEGs, DE-lncRNAs, and DE-miRNAs are indicated as ellipses, rectangular, and hexagon respectively. The red color represents the up-regulated expression, while the green represents the down-regulated expression.

As mentioned above, there were 82 potential DElncRNA–DEG (including 53 DE-lncRNAs and 78 DEGs, **Supplementary Table 10**), 30 potential DEmiRNA–DEG (including 8 DE-miRNAs and 23 DEGs, **Supplementary Table 11**), and 3 potential DEmiRNA–DElncRNA (including 3 DE-miRNAs and 2 DE-lncRNAs, **Supplementary Table 12**) interactions. Among them, the majority were potential one-to-one interactions, while the rest were potential one-to-many interactions, such as LNC_014758, which is predicted to regulate three up-regulated genes (*Cla013358*, *Cla013360*, *Cla013371*) and one down-regulated gene *Cla013363* (**Supplementary Figure 5**). Moreover, we only identified two potential miRNA–lncRNA–mRNA interactions (**Figure 4**). Compared to the Cl-miR171a–LNC_004095–Cla011387 interaction, the key regulatory network was much more complex, containing three DE-miRNAs (up-regulated nove_71 and down-regulated Cl-miR396a-5p and Cl-miR396b-5p), two DE-lncRNAs (down-regulated LNC_011135 and LNC_035385), and 21 DEGs including MYB transcription factor *Cla007663* and GROWTH REGULATING FACTOR (GRF) *Cla016859*. Among these DEGs, one up-regulated gene (*Cla014800*) and four down-regulated genes (*Cla010475*, *Cla012139*, *Cla013727*, and *Cla016859*) were predicted to be regulated by both Cl-miR396a-5p and Cl-miR396b-5p (**Figure 4**). Additionally, the down-regulated LNC_011135 was also detected as a potential target of two miRNAs, which was predicted to be regulated by *Cla016682* expression; however, the other down-regulated LNC_035385 was predicted to regulate three DEGs, including the genes *Cla016358* and *Cla016362* with decreased transcriptional abundance as well as a presumable target (*Cla016373*) of Cl-miR396b-5p with an increased transcript level.

### Identification of transcription factors possibly involved in male sterility

**Figure 5:**
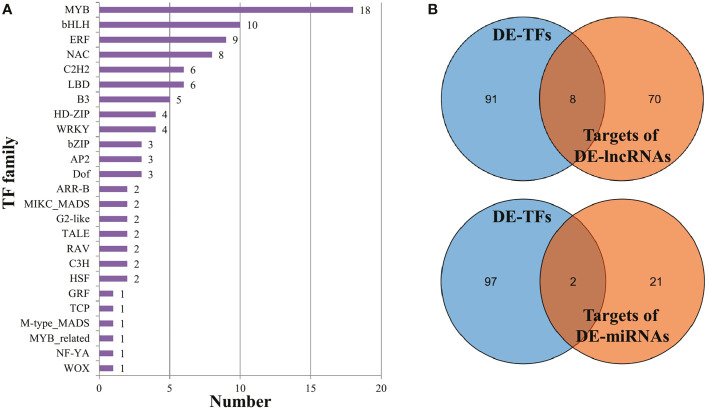
Transcription factors annotated in DEGs. **(A)** The members in each TF family. **(B)** The intersection of DE-TFs and targets of DE-lncRNAs (or DE-miRNAs).

Based on the plant transcription factor database PlantTFDB, approximately 54 up-regulated and 45 down-regulated DEGs were annotated as transcription factors, belonging to 25 families (**Figure 5A**). The MYB TF family, the largest one among them, contained 18 members, followed by bHLH (10), ERF (9), and NAC (8). Additionally, the majority of MYB TFs (15 out of 18) were enriched in the KEGG pathway ‘Plant-pathogen interaction’ (ko04626) (**Supplementary Table 8**). Combined with the analyses of DE-lncRNAs and DE-miRNAs, eight TFs were predicted to be regulated by DE-lncRNAs, and two TFs were possibly targeted by DE-miRNAs (**Figure 5B**). Notably, the bHLH TF *Cla002749* and GRF TF *Cla016859* were predicted to be regulated by two DE-lncRNAs and DE-miRNAs, respectively (**Supplementary Table 14**).

### DEGs involved in the phenylpropanoid biosynthesis and its related metabolism pathways

**Figure 6:**
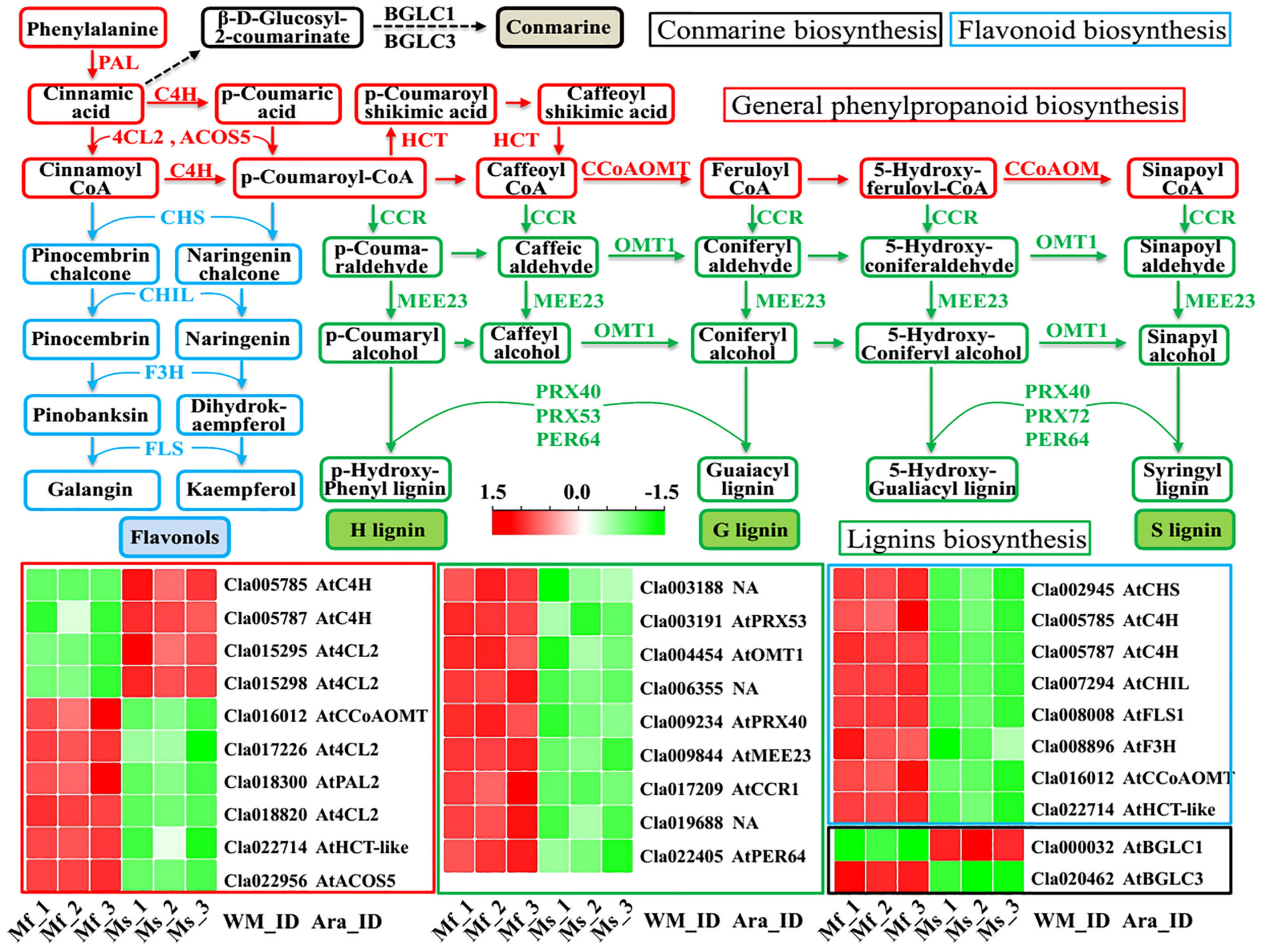
The general phenylpropanoid biosynthesis and its related secondary metabolisms enriched by KEGG analysis. Lines in different colors are indexed as followed: Red, general phenylpropanoid biosynthesis; Blue, chalcone and flavonol biosynthesis; Green, lignins biosynthesis; Black, conmarine biosynthesis.

As revealed by previous studies (Vogt, 2010; Yadav et al., 2020; Wei et al., 2021), the phenylpropanoid biosynthesis pathway, serving as a major specialized metabolism in plants, can provide various components for pollen and anther development, including coumarins, flavonoids, and lignins. According to the KEGG pathway analysis (**Supplementary Table 8**), phenylpropanoid biosynthesis (ko00940), along with phenylalanine metabolism (ko00360) and flavonoid biosynthesis (ko00941), was significantly enriched among the 1035 DEGs. For example, the *PAL* gene (*Cla018300*) encoding the enzyme phenylalanine ammonia lyase, with the function of catalyzing the amino acid phenylalanine into *trans*-cinnamic acid (**Figure 6**), was significantly down-regulated in male-sterile floral buds (log_2_(FC) = -1.01, **Supplementary Table 6**) and was also detected with considerably decreased abundance at protein level (Wei et al., 2021). Moreover, the accumulations of other key genes in the general phenylpropanoid biosynthesis pathway, e.g., *C4H* (*Cla005785* and *Cla005787*), *4CL2* (*Cla015295*, *Cla015298*, *Cla017226*, and *Cla018820*), *ACOS5* (*Cla022956*), *HCT* (*Cla022714*), *CCoAOMT* (*Cla016012*), were significantly altered in the male-sterile mutant Se18 (**Figure 6**). The intermediate CoA esters from the general phenylpropanoid biosynthesis pathway are subsequently synthesized into various lignins, *via* the actions of the enzymes CCR1 (Cla017209), MEE23 (Cla009844), OTM1 (Cla004454), PRX40 (Cla009234), PRX53 (Cla003191), and PER64 (Cla022405). As important intermediate constituents, CoA esters can be further transmitted into the flavonoid biosynthesis pathway (Falcone Ferreyra et al., 2012; Fellenberg and Vogt, 2015; Shi et al., 2015), in which cinnamoyl CoA and p-coumaroyl-CoA are catalyzed into pinocembrin chalcone and naringenin chalcone by chalcone synthase CHS (*Cla002945* with a -11.50-fold mRNA level in mutant Se18), respectively. As expected, the genes encoding enzymes that convert chalcones into the flavonols galangin and kaempferol were significantly down-regulated, including *CHIL* (*Cla007294*, -1.10-fold), *F3H* (*Cla008896*, -5.43-fold), and *FLS* (*Cla008008*, -2.81-fold) (**Figure 6**, **Supplementary Table 6**). Collectively, the conserved general phenylpropanoid biosynthesis pathway, as well as its related metabolism pathways, was inferred to undergo considerable disruption during another development in the male-sterile mutant Se18.

### DEGs involved in regulatory cascade in the nucleus

**Figure 7:**
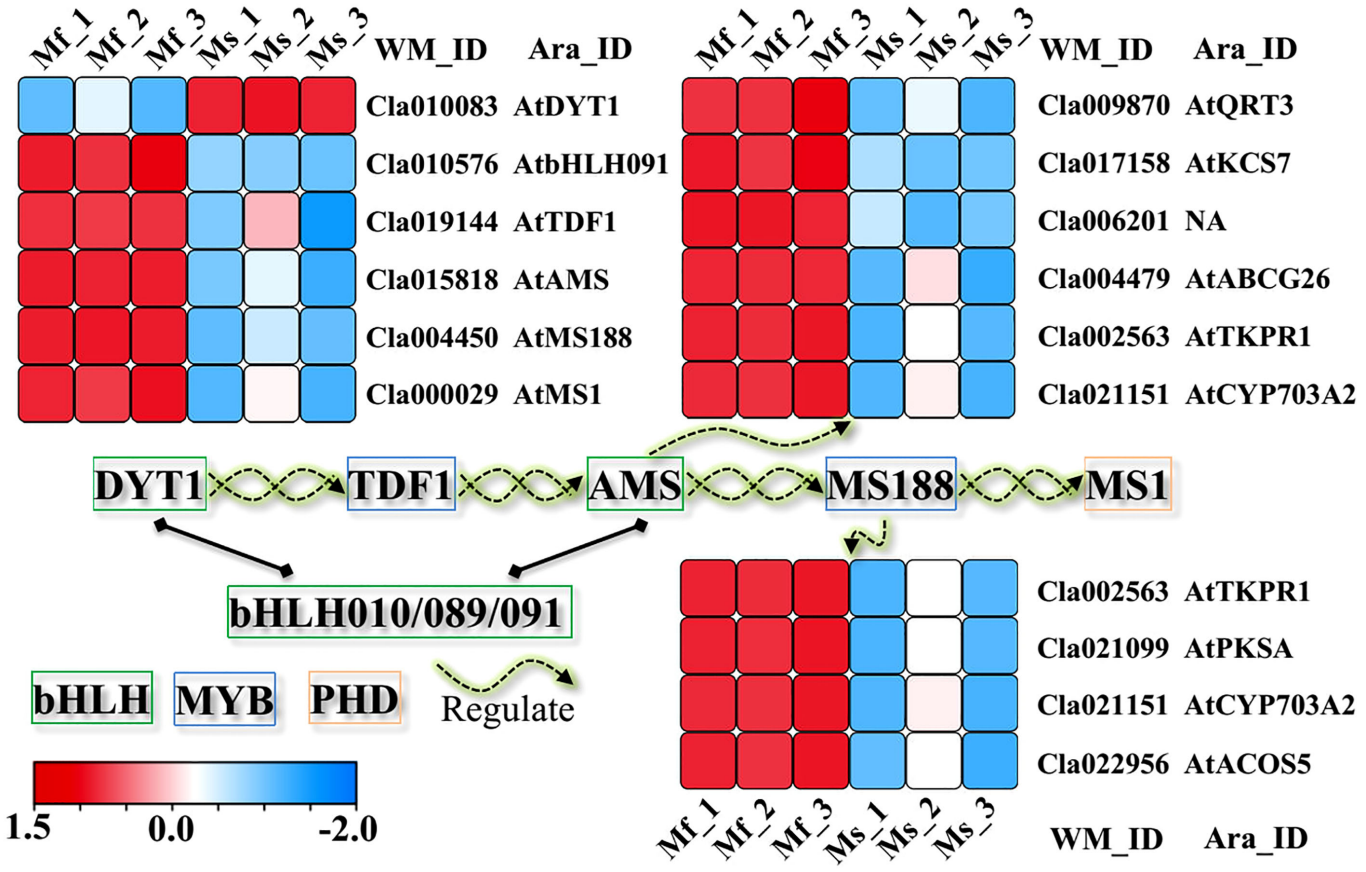
The disrupted genetic regulatory cascade in controlling tapetum development. All the TFs as well as the downstream targets of *AMS* (Xu et al., 2014) and *MS188* (Wang et al., 2018), were down-regulated in floral buds of mutant Se18, except for gene *DYT1* (*Cla010083*).

The transcriptional cascade *DYT1*–*TDF1*–*AMS*–*MS188*–*MS1* is considered to precisely regulate tapetum development and mature pollen production in plants (Wilson et al., 2001; Sorensen et al., 2003; Zhang, 2006; Zhang et al., 2007; Zhu et al., 2008; Jeong et al., 2014; Lu et al., 2020). Previously, three other bHLH TFs (bHLH010, bHLH089, bHLH091) were thoroughly characterized to function in pollen fertility, through interacting with DYT1 and/or AMS to activate downstream targets (Cui et al., 2016; Ferguson et al., 2017). In the watermelon male-sterile mutant Se18, the causal gene *ClATM1* (*Cla010576*) was recently cloned and identified as the ortholog of *Arabidopsis bHLH010*, *bHLH089*, and *bHLH091*, which is down-regulated in male-sterile flower of Se18 (Zhang et al., 2021), consistent with our transcriptome data (**Figure 7**). Meanwhile, all the other TFs in the regulatory cascade were decreased at a transcriptional level in mutant Se18, except for the gene *DYT1* (*Cla010083*). Functioning as the key regulators in this pathway (Xu et al., 2014; Wang et al., 2018; Lu et al., 2020), some targets of *AMS* and *MS188* were also down-regulated at the transcriptional level, such as *QRT2* (*Cla009870*), *ABCG26* (*Cla004479*), *ACOS5* (*Cla022956*), *CYP703A2* (*Cla021151*), *PKSA* (*Cla021099*), and *TKPR1* (*Cla002563*), demonstrating the obvious disruption of this conserved regulatory cascade in Se18.

## Discussion

Male flower development is a complex biological process regulated by precise networks, and dysfunction of any controlling genes possibly leads to male sterility. For example, a 10-bp sequence deletion in the bHLH gene *ClATM1* resulted in a complete male-sterile phenotype of the watermelon mutant Se18 (Zhang et al., 2021). Furthermore, according to our transcriptomic and TMT-based proteomic analyses, lipid metabolism, as well as the phenylpropanoid pathway, is predicted to be altered in mutant Se18 compared to its fertile progenitor (Wang et al., 2020; Wei et al., 2021). In the present study, the general phenylpropanoid pathway, together with its related metabolisms, was also impaired in male-sterile floral buds, as expected (**Figure 6**, **Supplementary Table 8**). Importantly, 14 out of the 25 DEGs enriched in phenylpropanoid-related pathways were also detected to have significant abundance changes at the protein level (**Supplementary Table 6**), further confirming the disruption of the corresponding metabolisms in male-sterile flower development. Given that the general phenylpropanoid pathway, together with its related secondary metabolisms, produces numerous substances, serving as important components for pollen wall development (e.g., lignin, coumarins, stilbenes, and flavonoids) (Vogt, 2010; Fellenberg and Vogt, 2015; Shi et al., 2015; Yadav et al., 2020; Wei et al., 2021), it is not surprising to detect substantial differences in plants with male reproductive defects, such as autotetraploid watermelon (Yi et al., 2022), tomato (Yang et al., 2019), *Brassica campestris* (Liang et al., 2019; Tang et al., 2022), cotton (Li et al., 2019a), and alfalfa (Xu et al., 2022).

In plants, non-coding RNAs (ncRNAs), including lncRNAs and miRNAs, have been determined to play essential roles in male reproductive development (Li et al., 2015). For example, the 828-bp lncRNA *BcMF11* from Chinese cabbage has been reported to function in pollen fertility (Song et al., 2013). Similarly, in hybrid rice, the sufficient abundance of the lincRNA LDMAR (1236 nt in length) is required for normal pollen formation under long-day conditions (Ding et al., 2012). In contrast with lncRNAs, miRNAs are endogenous single-stranded ncRNAs of 21 to 25 nt in length that function as post-transcriptional regulators in another development. For example, the conserved miR156 can directly target SPL genes to maintain anther fertility (Wang et al., 2009), while miR159 negatively regulates MYB members (e.g., *MYB33* and *MYB65*) to affect male sterility (Millar and Gubler, 2005). Recently, using high-throughput sequencing technologies, numerous candidate lncRNAs, as well as miRNAs, have been identified with potential roles in flower development, such as in tomato (Yang et al., 2019), *Brassica campestris* (Liang et al., 2019), cotton (Nie et al., 2018; Li et al., 2019a), and maize (Li et al., 2019b). In the present study, we identified a total of 80 DE-lncRNAs and 10 DE-miRNAs, which were predominantly down-regulated in the male-sterile mutant Se18 (**Figure 2**, **Supplementary Tables 9**, **11**). Consistently, the lipid- and phenylalanine-related metabolisms were also enriched, according to the KEGG analysis of genes potentially targeted by DE-lncRNAs (**Supplementary Table 8**). The interaction between evolutionarily conserved miR396 and its target GRF genes has been confirmed to be involved in plant development during vegetative and reproductive stages (Li et al., 2015; Yuan et al., 2020). Additionally, overexpression of miR396 was sufficient to induce pollen sterility (Yuan et al., 2020). However, in the watermelon mutant Se18, two miR396 members (Cl-miR396a and Cl-miR396b) were both detected to have decreased accumulations in male-sterile floral buds (**Supplementary Table 11**). Additionally, the two Cl-miR396 members, together with other DE-miRNAs, DE-lncRNAs, and DEGs, were predicted to form a complex miRNA–lncRNA–mRNAs regulatory regulatory network (**Figure 4**), to participate in male reproductive development in watermelon. Similar to the complex ceRNA networks in watermelon after cucumber green mottle mosaic virus (CGMMV) infection (Sun et al., 2020), diverse expression trends were also observed in this research (**Figure 4**, **Supplementary Figure 5**). For example, both Cl-miR396a and Cl-miR396b were down-regulated in male-sterile floral buds, whereas some of their predicted targets (e.g., *Cla014800* and *Cla016682*) were up-regulated, though others were down-regulated, including *Cla012139* and *Cla016859*. These results suggested the complexity of the ceRNA networks involved in male reproductive development in watermelon.

In plants, several transcription factors have been reported to be associated with tapetal function and pollen development, including two bHLH genes (*DYT1* and *AMS*), two MYB genes (*TDF1* and *MS188*) and one PHD gene (*MS1*) (Jeong et al., 2014; Nan et al., 2017). In addition, the proteins bHLH010, bHLH089, and bHLH091 have been confirmed to interact with DYT1 and AMS, respectively (Xu et al., 2010; Cui et al., 2016), and further activate *DYT1* through feed-forward and positive feedback loops (Cui et al., 2016). Based on the present transcriptome analyses, only *DYT1* (*Cla010083*) was detected with up-regulated expression in the watermelon mutant Se18, while the other four TFs were down-regulated (**Figure 7**, **Supplementary Table 6**). In addition, *AMS* and *MS188* function as key regulators (Xu et al., 2014; Wang et al., 2018; Lu et al., 2020), some targets of which were also expected to be decreased in mutant male-sterile flower buds, including *QRT2* (*Cla009870*), *ABCG26* (*Cla004479*), *ACOS5* (*Cla022956*), *CYP703A2* (*Cla021151*), *PKSA* (*Cla021099*), and *TKPR1* (*Cla002563*). Furthermore, approximately 99 differentially expressed TFs were identified, belonging to 25 families (**Supplementary Table 14**). Notably, the bHLH transcription factor *Cla002749* and GRF TF *Cla016859* were predicted to be regulated by two DE-lncRNAs and DE-miRNAs respectively, providing valuable candidates for further research. Previously, *ClAMT1*, the causal gene for male sterility in Se18, was functionally characterized (Zhang et al., 2021), revealing it displays a clear dose effect and down-regulation in the Se18 mutant, consistent with the transcriptome data (**Figure 7**). However, we did not detect any miRNAs or lncRNAs that regulate *ClAMT1* according to our bioinformatic analyses. Thus, this study offers new insights into the regulatory mechanisms underlying male sterility in watermelon and provides valuable candidates for further exploration of the complex regulatory networks.

## Data availability statement

The data presented in the study are deposited in the NCBI database repository, accession number PRJNA926906.

## Author contributions

YZ, PXN: Conceptualization. YL, ZRM, WCH: Methodology. LJY, SFF, YLJ: Software. HYJ, CXY, LX: Validation. YZ, PXN: Formal analysis. ZY, MJX: Investigation. YJQ, LFS: Resources. YZ: Roles/Writing—original draft. LH, ZRM, WCH: Writing & editing. WCH: Visualization. YL, ZRM, WCH: Supervision. HWF, ZX, WCH: Funding acquisition. All authors contributed to the article and approved the submitted version.

## Supplementary material

Supplementary Figure 1 Pearson correlation analyses of transcriptome **(A)** and small RNA **(B**) sequencing data among biological replicates. The color scale indicates the strength of the correlation, and the deeper color reflects higher correlation.

Supplementary Figure 2 Detail information of lncRNAs. **(A)** The basic identification steps for lncRNAs. **(B)** Screening non-coding lncRNAs using three databases (CPC2, CNCI, and PFAM). **(C)** Classification of lncRNAs. Comparison of exon number **(D)** and length **(E)** between mRNAs and lncRNAs.

Supplementary Figure 3 Comparative and GO enrichment analyses of DEGs. **(A)** The abundance of DEGs at transcriptional and protein levels. Brief schematic diagram of GO terms of DEGs in BP category **(B),** as well as differentially expressed *cis*-targets of DE-lincRNAs in MF **(C)** and BP **(D)** categories. GO ID, term description, *p*-adjust value, genes in input and background lists were shown in each term.

Supplementary Figure 4 Detailed GO terms of DEGs in BP category. The detailed information of GO terms was displayed in following order: GO term, term description, *p*-adjust value, genes in input and background lists. The colors represented the level of significance in each term.

Supplementary Figure 5 Potential regulatory networks of DEGs, DE-lncRNAs, and DE-miRNAs. DEGs, DE-lncRNAs, and DE-miRNAs are indicated as ellipses, rectangular, and hexagon respectively. The red color represents the up-regulated expression, while the green represents the down-regulated expression.
