# Supplementary figures and images for: Whole-transcriptome analyses identify key differentially expressed mRNAs, lncRNAs, and miRNAs associated with male sterility in watermelon
Source: Front Plant Sci. 2023 Mar 2;14:1138415. doi: 10.3389/fpls.2023.1138415 (PMC10019506; doi:10.3389/fpls.2023.1138415)

**a**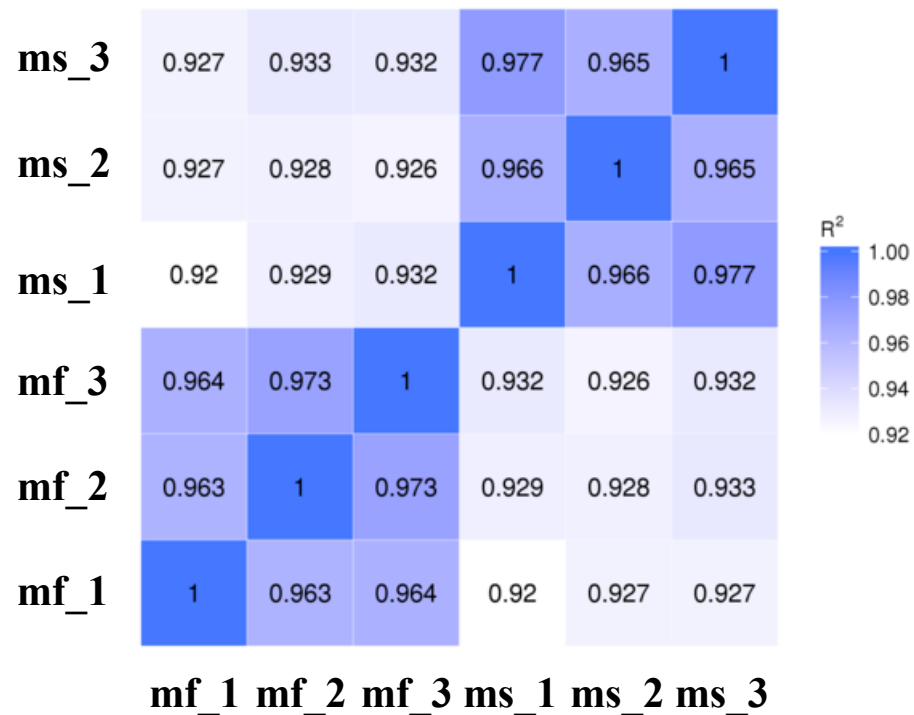**b**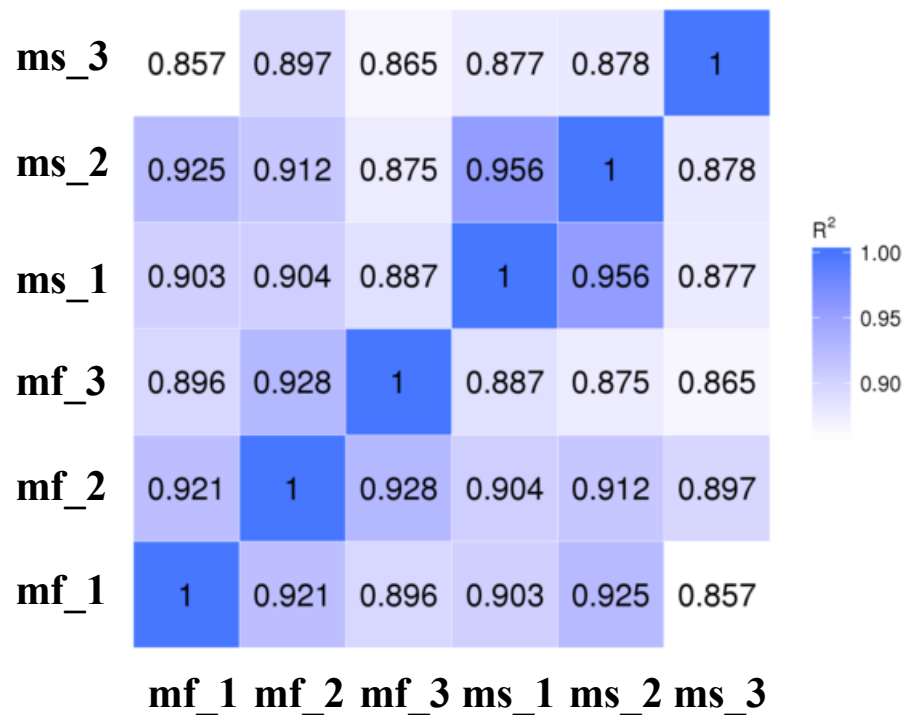

Supplement: Supplementary Figure 1 — Pearson correlation analyses of transcriptome (A) and small RNA (B) sequencing data among biological replicates. The color scale indicates the strength of the correlation, and the deeper color reflects higher correlation. [file DataSheet_1.pdf]

**a**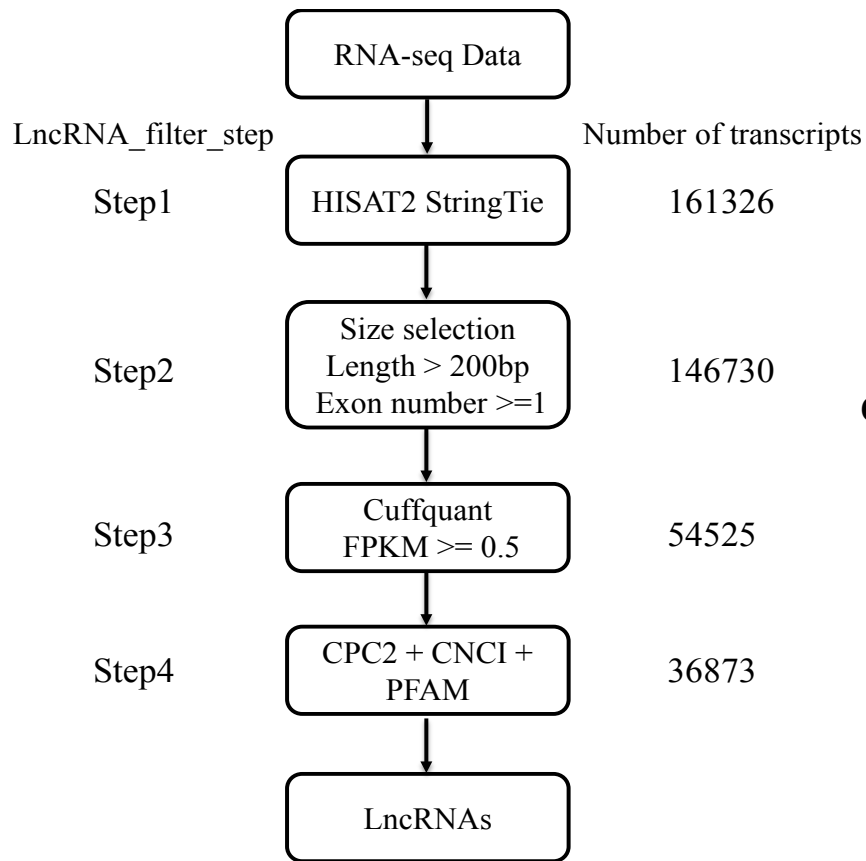**b**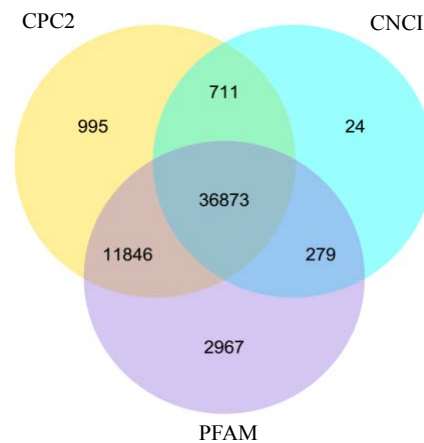**c**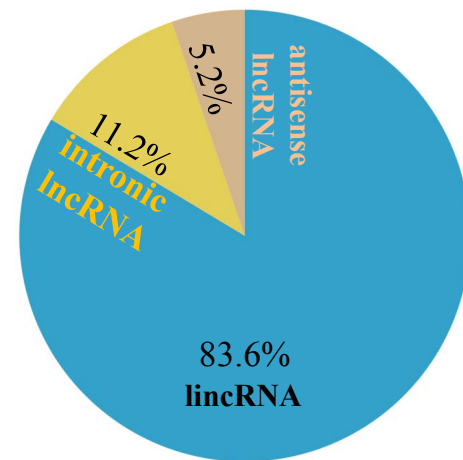**d**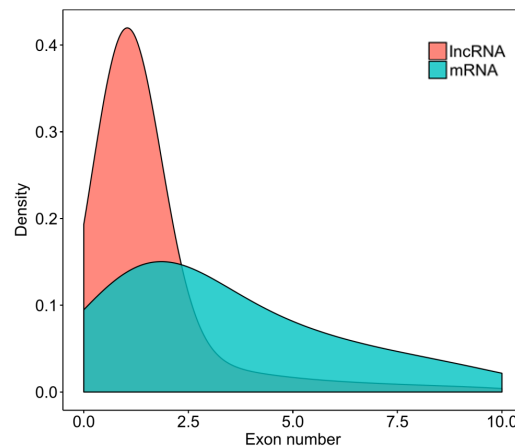**e**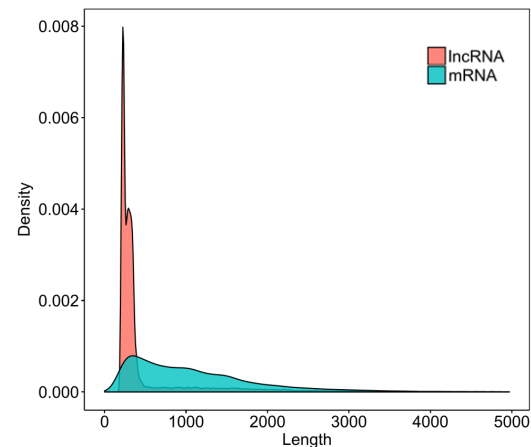

Supplement: Supplementary Figure 2 — Detail information of lncRNAs. (A) The basic identification steps for lncRNAs. (B) Screening non-coding lncRNAs using three databases (CPC2, CNCI, and PFAM). (C) Classification of lncRNAs. Comparison of exon number (D) and length (E) between mRNAs and lncRNAs. [file DataSheet_2.pdf]

a

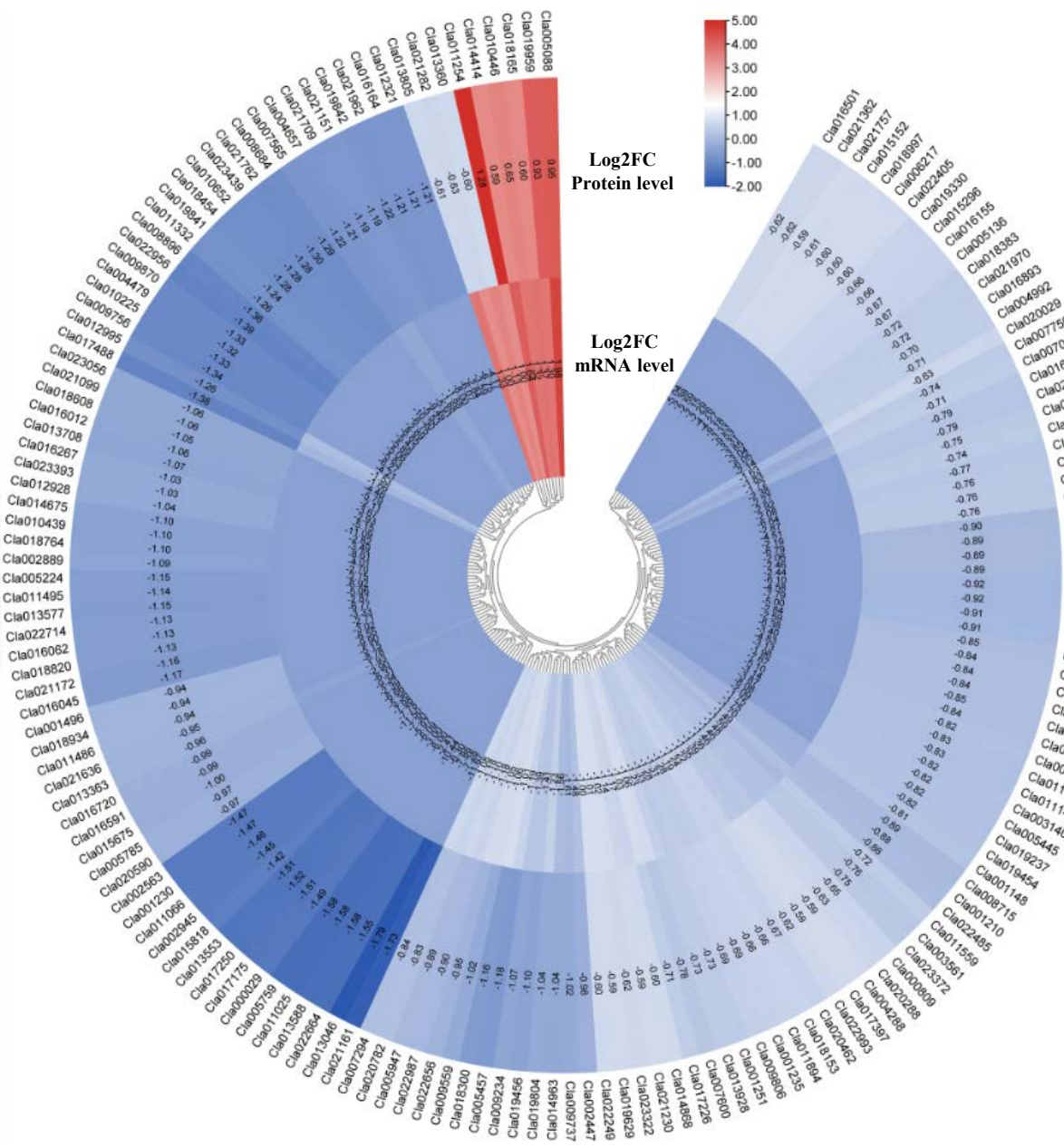

b

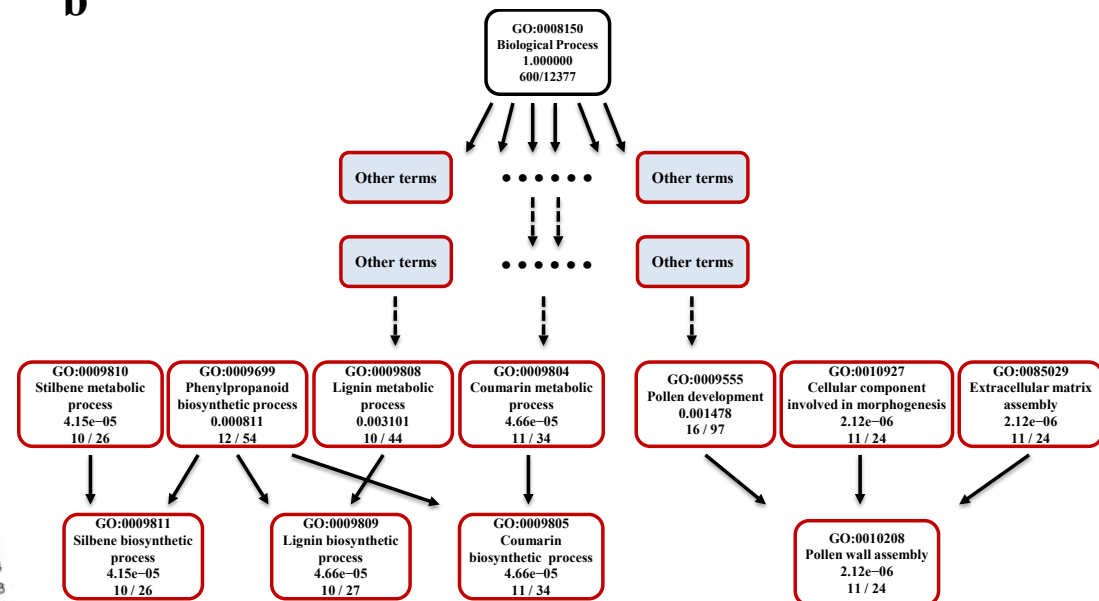

c

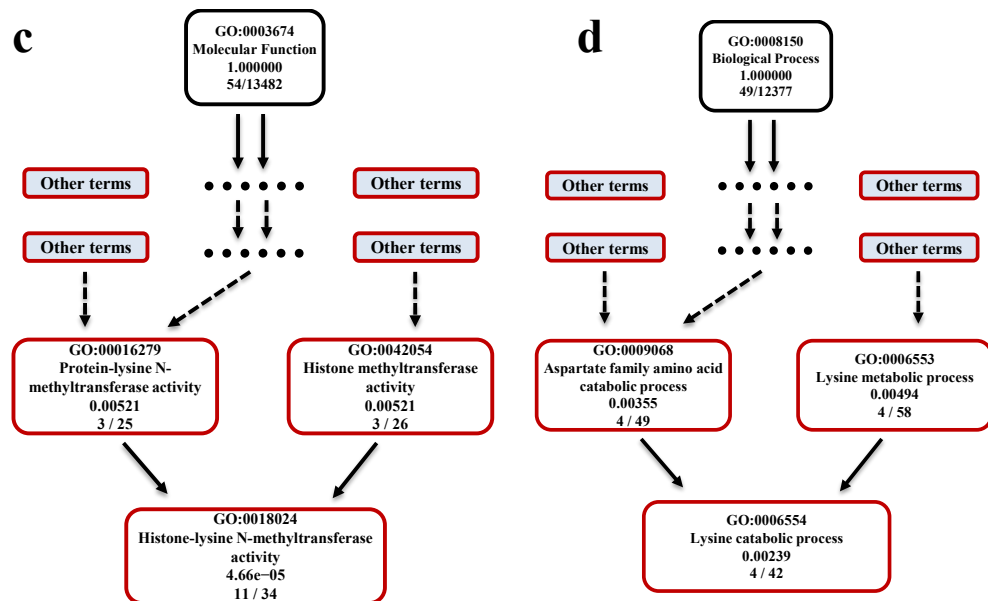

d

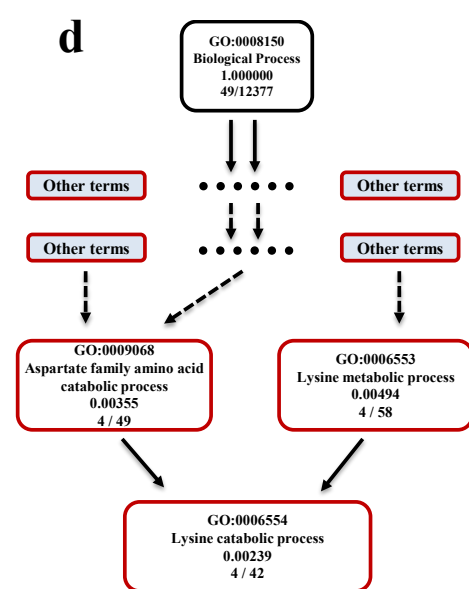

Supplement: Supplementary Figure 3 — Comparative and GO enrichment analyses of DEGs. (A) The abundance of DEGs at transcriptional and protein levels. Brief schematic diagram of GO terms of DEGs in BP category (B), as well as differentially expressed cis-targets of DE-lincRNAs in MF (C) and BP (D) categories. GO ID, term description, p-adjust value, genes in input and background lists were shown in each term. [file DataSheet_3.pdf]

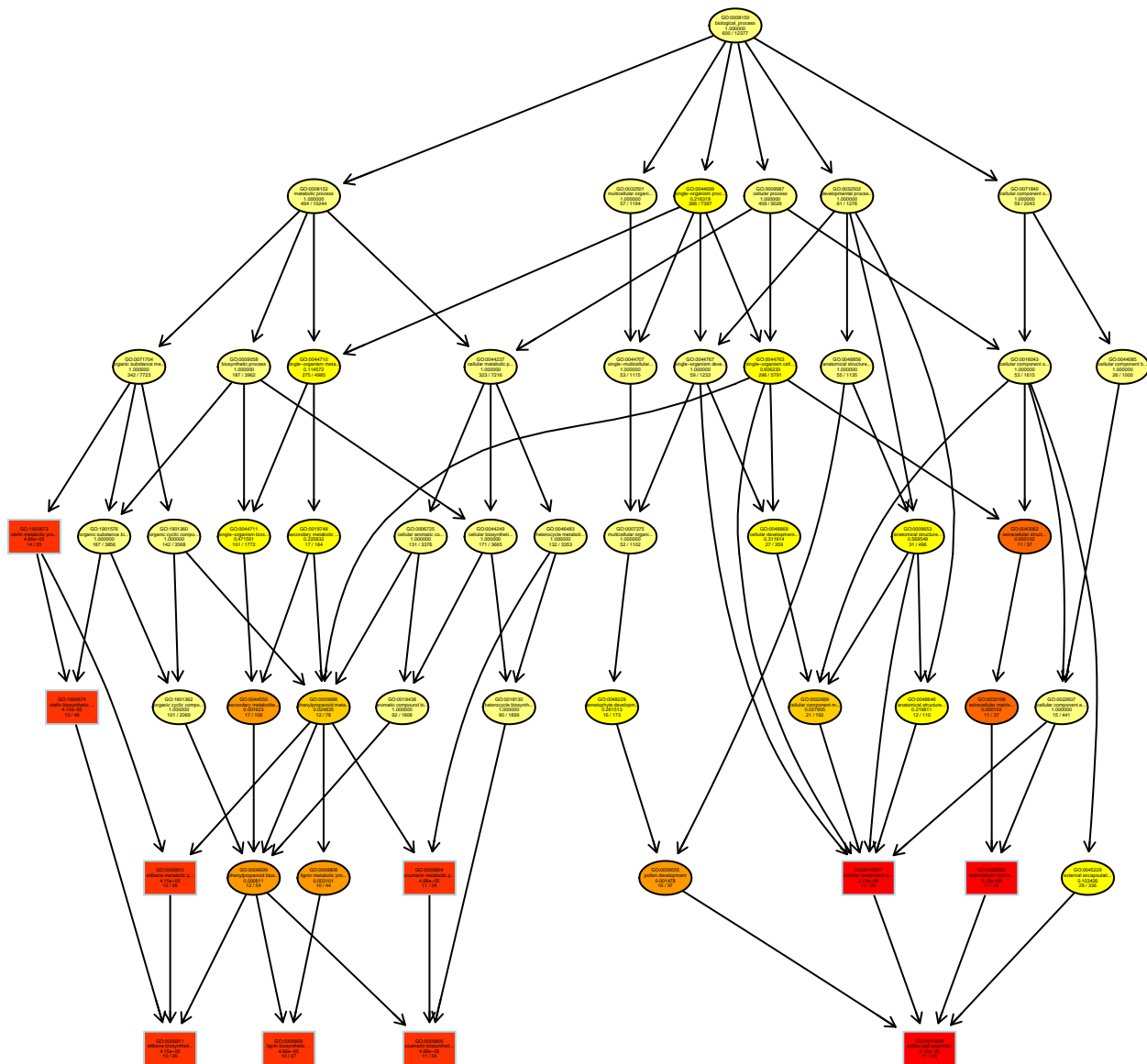

Supplement: Supplementary Figure 4 — Detailed GO terms of DEGs in BP category. The detailed information of GO terms was displayed in following order: GO term, term description, p-adjust value, genes in input and background lists. The colors represented the level of significance in each term. [file DataSheet_4.pdf]

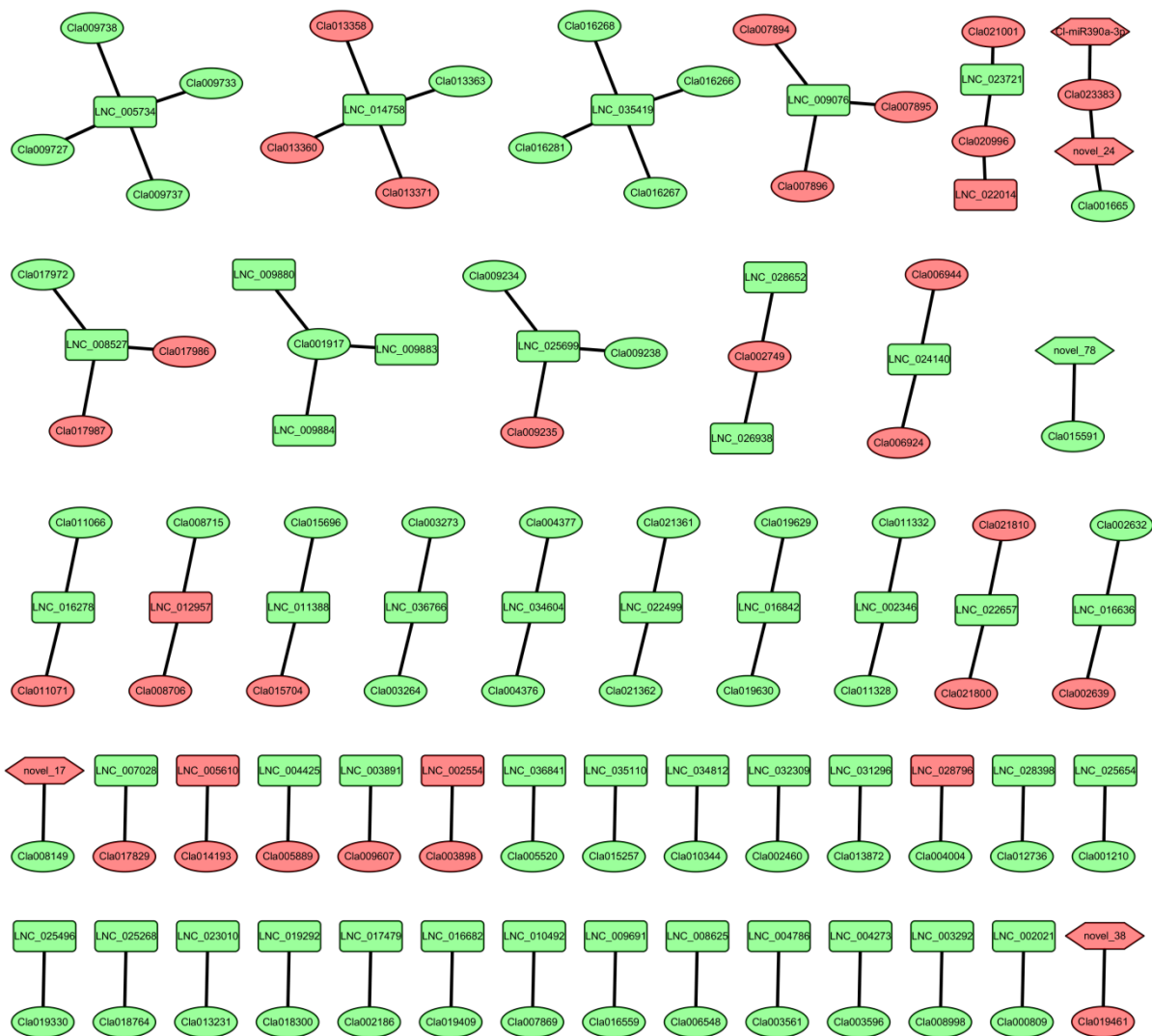

Supplement: Supplementary Figure 5 — Potential regulatory networks of DEGs, DE-lncRNAs, and DE-miRNAs. DEGs, DE-lncRNAs, and DE-miRNAs are indicated as ellipses, rectangular, and hexagon respectively. The red color represents the up-regulated expression, while the green represents the down-regulated expression. [file DataSheet_5.pdf]
